# Biochanin A Regulates Key Steps of Inflammation Resolution in a Model of Antigen-Induced Arthritis via GPR30/PKA-Dependent Mechanism

**DOI:** 10.3389/fphar.2021.662308

**Published:** 2021-04-26

**Authors:** Franciel Batista Felix, Juliana Priscila Vago, Débora de Oliveira Fernandes, Débora Gonzaga Martins, Isabella Zaidan Moreira, William Antonio Gonçalves, Walyson Coelho Costa, Jessica Maria Dantas Araújo, Celso Martins Queiroz-Junior, Gabriel Henrique Campolina-Silva, Frederico Marianetti Soriani, Lirlândia Pires Sousa, Renata Grespan, Mauro Martins Teixeira, Vanessa Pinho

**Affiliations:** ^1^ Departamento de Morfologia, Instituto de Ciências Biológicas, Universidade Federal de Minas Gerais, Belo Horizonte, Brazil; ^2^ Departamento de Análises Clínicas e Toxicológicas, Faculdade de Farmácia, Universidade Federal de Minas Gerais, Belo Horizonte, Brazil; ^3^ Departamento de Fisiologia, Universidade Federal de Sergipe, São Cristovão, Brazil; ^4^ Departamento de Genética, Ecologia e Evolução, Instituto de Ciências Biológicas, Universidade Federal de Minas Gerais, Belo Horizonte, Brazil; ^5^ Departamento de Bioquímica e Imunologia, Instituto de Ciências Biológicas, Universidade Federal de Minas Gerais, Belo Horizonte, Brazil

**Keywords:** biochanin A (PubChem CID 5280373), arthritis, apoptosis, efferocytosis, Resolution of inflammation

## Abstract

Biochanin A (BCA) is a natural organic compound of the class of phytochemicals known as flavonoids and isoflavone subclass predominantly found in red clover (*Trifolium pratense*). It has anti-inflammatory activity and some pro-resolving actions, such as neutrophil apoptosis. However, the effect of BCA in the resolution of inflammation is still poorly understood. In this study, we investigated the effects of BCA on the neutrophilic inflammatory response and its resolution in a model of antigen-induced arthritis. Male wild-type BALB/c mice were treated with BCA at the peak of the inflammatory process (12 h). BCA decreased the accumulation of migrated neutrophils, and this effect was associated with reduction of myeloperoxidase activity, IL-1β and CXCL1 levels, and the histological score in periarticular tissues. Joint dysfunction, as seen by mechanical hypernociception, was improved by treatment with BCA. The resolution interval (Ri) was also quantified, defining profiles of acute inflammatory parameters that include the amplitude and duration of the inflammatory response monitored by the neutrophil infiltration. BCA treatment shortened Ri from ∼23 h observed in vehicle-treated mice to ∼5.5 h, associated with an increase in apoptotic events and efferocytosis, both key steps for the resolution of inflammation. These effects of BCA were prevented by H89, an inhibitor of protein kinase A (PKA) and G15, a selective G protein–coupled receptor 30 (GPR30) antagonist. In line with the *in vivo* data, BCA also increased the efferocytic ability of murine bone marrow–derived macrophages. Collectively, these data indicate for the first time that BCA resolves neutrophilic inflammation acting in key steps of the resolution of inflammation, requiring activation of GPR30 and via stimulation of cAMP-dependent signaling.

## Introduction

Arthritis is a severe inflammatory disease characterized by synovial inflammation, production of inflammatory mediators, intense pain, destruction of cartilage and bone, and systemic dysfunctions (Firestein, 2003; McInnes and Schett, 2017; Smolen et al., 2018). Neutrophils are the main inflammatory cells recruited to the inflamed joint and have a significantly extended life span, contributing to the amplification of the inflammatory reaction, pain, and tissue damage (Wright et al., 2014; Fattori et al., 2016; Cecchi et al., 2018). Previous studies have shown that failure to resolve ongoing inflammation in arthritis may be the major contributor to persistent and chronic inflammation (Perretti et al., 2017; Schett, 2019).

The resolution of inflammation is a complex process and actively orchestrated by specialized pro-resolving mediators that collaborate efficiently to inhibit the influx of leukocytes to the inflamed site, shutting down molecules of intracellular pathways associated with cytokine production and leukocyte survival (especially neutrophils) (Serhan and Savill, 2005; Alessandri et al., 2013; Fullerton and Gilroy, 2016; Feehan and Gilroy, 2019), which eventually will lead to apoptosis of these cells and their subsequent removal by macrophages in a process named efferocytosis (Poon et al., 2014; Green et al., 2016; Morioka et al., 2019; Lawrence et al., 2020). These steps lead to the resolution of inflammation and tissue repair and represent the potential therapeutic target for resolution pharmacology (Martin et al., 2015; Perretti et al., 2015; Greenlee-Wacker, 2016; Sugimoto et al., 2016). In arthritis, a failed neutrophil apoptosis and an impaired efferocytosis are correlated with the severity of the disease (Abdolmaleki et al., 2018).

Biochanin A (5,7-dihydroxy-4′-methoxyisoflavone, BCA) is a natural organic compound of the class of phytochemicals known as flavonoids and isoflavone subclass predominantly found in red clover (*Trifolium pratense*), legume plants, and many other herbal products (Yu et al., 2019; Sarfraz et al., 2020). BCA has a remarkably close structural resemblance with endogenous estrogen 17β-estradiol (E2) (Raheja et al., 2018; Křížová et al., 2019) and has been reported to act via nuclear estrogen receptors (ERs, *α* and β) and membrane-bound ERs, such as G protein–coupled receptor 30 (GPR30, also known as G protein–linked estrogen receptor 1) (Suetsugi et al., 2003; Molina et al., 2018). GPR30 is a common target for isoflavones to promote a rapid cellular signaling (Khan et al., 2015; Liu et al., 2018), and it is expressed in the central nervous system, skin, adipose tissue, and skeletal muscle, as well as in monocytes, eosinophils, and neutrophils (Rettew et al., 2010; Notas et al., 2020). Furthermore, GPR30 expression was also observed in cartilage and bone during an inflammatory response (Kang et al., 2015; Ribeiro et al., 2020; Zhao et al., 2020). Activation of GPR30 results in increased intracellular levels of cyclic adenosine monophosphate (cAMP) and activates the PKA signaling pathway, the best known cAMP effector (Mizukami, 2010; Liu et al., 2018) and an essential pathway for a successful inflammation resolution (Lima et al., 2017; Negreiros-Lima et al., 2020; Tavares et al., 2020). Current findings have demonstrated that increased levels of cAMP by selective inhibition of phosphodiesterase 4 (PDE4), an enzyme that metabolizes cAMP, lead to neutrophil apoptosis, enhanced clearance of dead cells by efferocytosis, and complete resolution of inflammation in a model of lipopolysaccharide-induced pleurisy (Sousa et al., 2010; Lima et al., 2017).

BCA is known for a wide spectrum of pharmacological characteristics, mainly for its anti-inflammatory effects during arthritis and osteoporosis (Felix et al., 2020; Liao et al., 2021), asthma(Ko et al., 2011), and neuropathic pain (Chundi et al., 2016), by inhibiting pro-inflammatory signaling and transcription factors, such as reducing the activation of NF-κB (nuclear factor kappa beta) and blocking the levels of TNF-α (tumor-necrosis factor-alpha), IL-1β (interleukin 1 beta), and IL-2. In addition, the antitumor effect of BCA is correlated with its ability to stimulate cell apoptosis (Puthli et al., 2013; Hsu et al., 2018). Recently, we showed that BCA induced neutrophilic apoptosis *in vitro* (Felix et al., 2020). However, BCA’s ability to influence pro-resolving pathways is currently lacking.

In this study, we challenge the hypothesis that alongside its recognized anti-inflammatory actions, BCA also possesses pro-resolving properties. Using a well-characterized model of antigen-induced arthritis (AIA), we demonstrate that therapeutic administration of BCA resolves neutrophilic inflammation acting in key steps of the resolution of inflammation, requiring activation of GPR30, and via stimulation of cAMP-dependent signaling.

## Materials and Methods

### Animals and Ethical Approval

Eight- to ten-week-old male BALB/c mice (20–25 g) were obtained from the animal facility of the Federal University of Minas Gerais. Animals were kept under 12-h light/dark cycles at 25°C and were given water and feed ad libitum. Experiments were carried in accordance with the recommendations of the National Council for Control of Animal Experimentation (CONCEA) and were approved under the protocol number 160/2019 by the Animal Ethics Committee of the Federal University of Minas Gerais (CEUA/UFMG).

### Antigen-Induced Arthritis

Antigen-induced arthritis was induced, as previously described (Lopes et al., 2011; Gonçalves et al., 2020). In brief, mice were placed under anesthesia (100 μL of a mixture of 100 mg/kg of ketamine and 15 mg/kg of xylazine, intraperitoneally) were immunized intradermally at the base of the tail with 500 μg of methylated bovine serum albumin (mBSA, Sigma Aldrich–St. Louis, MO, United States) dissolved in an emulsion containing 50 μL of phosphate-buffered solution (PBS) and 50 μL of complete Freund’s adjuvant (CFA; 1 mg/ml of *Mycobacterium tuberculosis*, Sigma Aldrich–St. Louis, MO, United States). To induce arthritis, 14 days after immunization, antigen challenge was performed by intra-articular injection of 10 µg of mBSA diluted in 10 µL of sterile PBS into the right tibiofemoral knee joint in anesthetized mice, while its contralateral joint was injected with an equal volume of PBS. Subsequently, mice were euthanized with an overdose of anesthesia (100 μL of a mixture of 180 mg/kg of ketamine and 24 mg/kg of xylazine, intraperitoneally) at different time points (2, 6, or 12 h) after the treatment, and the knee cavity was washed with PBS/BSA 3% (2 × 5 µL) to harvest cells. Total cell counts were determined in a Neubauer chamber using Turk’s stain, and differential leukocyte counts were determined using standard morphologic criteria and were performed on cytospin (Shandon III, Thermo Shandon, Frankfurt, Germany) slides stained with Panoptic Solutions (Laborclin, PR, Brazil). Periarticular tissue was removed from the joint for evaluation of cytokines, chemokines, and activities of N-acetylglucosaminidase (NAG) and myeloperoxidase (MPO).

### Treatment Protocols

BCA was dissolved, as previously described (Suliman et al., 2018). In brief, a stock solution of BCA (Sigma Aldrich–St. Louis, MO, United States) was prepared first in dimethylsulfoxide (DMSO), and then a working solution was obtained by mixing the stock solution with sterile PBS (1:9, DMSO: PBS). Initial experiments evaluated the optimal dose of BCA in the AIA model. Mice were treated intraperitoneally with 0.36, 1.8, and 9 mg/kg of the BCA 12 h after mBSA challenging (at the peak of inflammation). The dose of 9 mg/kg was found to be optimal for the resolution of inflammation and was used in all subsequent experiments. Control mice received only the drug vehicle.

To assess whether the effects of BCA require activation of GPR30 and PKA-dependent mechanism, mice received an intra-articular injection of G15 (50 μg, Cayman chemical, Ann Arbor, MI, United States), a GPR30 selective antagonist, and H89 (100 μg, Sigma Aldrich–St. Louis, MO, United States), a PKA inhibitor, 15 min before injecting BCA. To evaluate leukocyte apoptosis, zVAD-fmk (1 mg/kg, Tocris Bioscience), a broad-spectrum caspase inhibitor, was given intraperitoneally 15 min before BCA injection. Drugs were dissolved in DMSO and further diluted in PBS. These drug doses were based on previous studies (Dennis et al., 2009; Lima et al., 2017; Galvão et al., 2019a; Felix et al., 2020). Knee wash was performed 2, 6, or 12 h after treatment. Of note, treatments and experimental procedures were performed in a blinded manner.

### Quantification of Neutrophil and Macrophage Accumulation in Periarticular Tissue

Quantification of MPO and NAG activities (a quantitative measurement of neutrophil and macrophage sequestration, respectively) in periarticular tissue homogenates was evaluated by an enzymatic reaction, measured at 450 nm in a spectrophotometer, as described elsewhere (Barroso et al., 2017). Results are expressed as absorbance.

### Measurement of IL-1β and CXCL1

Periarticular tissue was collected and homogenized in a homogenizer (Quiagen, Biotecnology Brazil Ltda, São Paulo, SP, Brazil) for 5 min in a solution containing antiproteases, as previously described (Coelho et al., 2008). The samples were centrifuged for 10 min at 10,000 rpm at 4°C. The concentration of IL-1β and CXCL1 was measured by enzyme-linked immunosorbent assay (ELISA) in the supernatants of the homogenates and using commercially available antibodies according to the procedures supplied by the manufacturer (R&D Systems, Minneapolis, MN, United States). In brief, each well on the plate was coated with the capture antibody (diluted in PBS) and incubated overnight at 4°C. The plate was washed and blocked with 100 μL of blocking solution (BSA 1%) at 37°C for 1 h. After washing steps, 50 μL of sample (diluted 1:1 in BSA 0.1%) or standard curve was added per well and incubated overnight at 4°C. The plate was washed and incubated at 37°C for 2 h with 50 μL of biotinylated detection antibodies per well. After washing, the plate was incubated at 37°C for 20 min with streptavidin–HRP. And, 50 μL of TMB (tetramethylbenzidine) was added to each well after washing steps and incubated at 37°C. After the appropriate time, 50 μL of stop solution (H2SO4, 1 M) was added to each well, and the plates were read at 450 nm using a microplate reader (BioTek). Washing steps were performed four times with PBS–Tween 20 0.1%. All samples were analyzed in duplicate.

### Evaluation of Hypernociception

The mechanical hypernociception was evaluated, as previously described (Gonçalves et al., 2020). To identify the withdrawal threshold, we used an electronic von Frey algesimeter (Insight Instruments, Ribeirão Preto, SP, Brazil). The dorsiflexion-elicited withdrawal threshold was expressed in grams 7) and used to infer behavioral responses associated with experimental pain (hypernociception). The nociceptive response was measured 6 or 12 h after BCA treatment.

### Calculation of Resolution Indices

Resolution indices were calculated, as previously described (Bannenberg et al., 2005; Barroso et al., 2017). Synovial fluid was collected from articular cavity at 12, 18, 24, 48, and 72 h after mBSA challenge. BCA treatment was performed at the peak of inflammation, 12 h after the challenge. Total cell counts were determined in a Neubauer chamber, and differential leukocyte counts were determined using standard morphologic criteria on a slide stained with Panoptic Solutions. The resolution interval (R*i*) was calculated by kinetic local of neutrophil infiltration defined in quantitative terms by the following resolution indices: 1) magnitude: Ψ_max_ (the maximum neutrophil count that is present during the inflammatory response) and T_max_ (peak of neutrophil infiltration); 2) duration: T_50_ (time point when the neutrophil count reduces to 50% of maximum); and 3) resolution interval R*i* (the interval between T_max_ and T_50_, when 50% neutrophils are lost from the articular cavity).

### Assessment of Leukocyte Apoptosis

Apoptosis was assessed morphologically, as reported previously (Barroso et al., 2017). In brief, 5 × 10^4^ cells collected 18 h after mBSA challenge were cyto-centrifuged, fixed, and stained with May–Grünwald–Giemsa and counted using oil immersion microscopy (×100 objective) to determine the proportion of cells with distinctive apoptotic morphology in a blind manner. Of note, cells were considered apoptotic when they exhibited chromatin condensation, nuclear fragmentation, and formation of apoptotic bodies outside or inside of macrophages (Poon et al., 2014). At least 500 cells were counted/slide, and results are expressed as mean ± SEM of percentage of cells with apoptotic morphology. Apoptosis was also evaluated by flow cytometry (FACS Canto II, BD Biosciences). Then, mice were injected with mBSA, and 12 h later, locally treatment with BCA was performed. For the apoptosis assays, the lavage of the knee was performed 4 h after the treatment with drugs. Cells were surface-stained for 30 min with the anti-LY6G-BV421 antibody (eBioscience) and then labeled with annexin-V-APC and propidium iodide (PI), as an index of loss of nuclear membrane integrity (PE Annexin V Apoptosis Detection Kit; BD PharmingenTM; United States).

### Western Blot Analysis

Synovial tissue samples (20–40 mg tissue) were homogenized using cell lysis buffer (1% Triton X-100, 100 mM Tris/HCl, pH 8.0, 10% (v/v) glycerol, 5 mM EDTA, 200 mM NaCl, 1 mM DTT, 1 mM PMSF, 2.5 μg/ml leupeptin, 5 μg/ml aprotinin, 1 mM sodium orthovanadate), as previously reported (Galvão et al., 2019b). Protein amounts were quantified with the Bradford assay reagent from Bio-Rad (Bio-Rad, Hercules, CA, United States). Extracts (50 μg) were separated by electrophoresis on denaturing, 10% polyacrylamide-SDS gel and electrotransferred to nitrocellulose membranes (Hybond ECL, GE Healthcare). Membranes was incubated overnight at 4°C with specific primary antibodies for GPR30 (1:500, Millipore) or GAPDH (1:1,000, Cell Signaling) in PBS containing 5% (w/v) BSA and 0.1% Tween-20. After washing, the membranes were incubated with an appropriate HRP-conjugated secondary antibody (1:3,000). Immunoreactive bands were visualized by using an ECL detection system, as described by the manufacturer (GE Healthcare, Piscataway, NJ, United States).

### Thymocytes Preparation, Labeling, and Apoptosis Induction

Thymocytes were isolated from the thymus of 8-week-old BALB/c mice using established protocols (Zhen and Shao, 2019). In brief, thymocytes were labeled with carboxyfluorescein diacetate succinimidyl ester (CFSE, 10 μM, Life Technologies, Carlsbad, CA, United States). Next, CFSE-labeled thymocytes were stimulated with staurosporine (1 μM, Sigma-Aldrich) for the induction of apoptosis for 4 h at 37°C under light protection. The percentage of apoptosis was confirmed in a parallel aliquot of unlabeled thymocytes by flow cytometry (FACS Canto II, BD Biosciences) using annexin-V APC and PI) and >85% were apoptotic.

### Efferocytosis Assay *In Vivo*

This protocol is an adaptation of previously described protocols (Das et al., 2014; Newson et al., 2014), and it has been applied in previous publication of our group (Perez et al., 2019; Vago et al., 2019; Negreiros-Lima et al., 2020). Mice received an intraperitoneal (i.p.) injection of zymosan 0.1 mg/cavity. After 90 h, mice were treated with BCA (9 mg/kg, i.p.) for 6 h. G15 (50 µg) was given systemically (i.p.) 30 min before BCA injection. Then, after the treatment with BCA, mice received an i.p. injection containing apoptotic thymocytes labeled with CFSE (3 × 10^6^ cells/cavity). Mice were euthanized 30 min after injection of the apoptotic thymocytes, and cells were recovered from the peritoneal cavity and incubated for labeling with a fluorescent anti-F4/80-PE-Cy7 antibody for 20 min (eBioscience). The cells were analyzed by flow cytometry (FACS Canto II, BD Biosciences). The results of flow cytometry are presented as percentage of F4/80^+^/CFSE^+^ cells. Moreover, efferocytosis was also performed by preparing cytospin slides and determining the proportion of cells with efferocytic morphology (macrophage with apoptotic bodies observed in their cytoplasm), and 500 cells/slides were counted. Results are expressed as mean ± SEM of percentage of macrophages with apoptotic thymocytes inside.

### Neutrophil Isolation, Labeling, and Apoptosis induction

Neutrophils were purified from the peripheral blood of healthy donors, as described previously (Galvão et al., 2019a). In brief, blood was collected into ethylenediaminetetraacetic acid (EDTA) and was separated through a double-density gradient using Histopaque 10771 and 11191 (both Sigma-Aldrich). After polymorphonuclear cell isolation and wash, contaminating erythrocytes were removed by hypotonic lysis. Neutrophil isolates were approximately 96% pure as confirmed by morphological appearance using light microscopy and resuspended in the RPMI-1640 medium and incubated at 37°C in a 5% CO_2_ atmosphere. Thus, neutrophils were labeled with CFSE (10 μM) and stimulated with staurosporine (10 μM) for induction of apoptosis for 1 h at 37°C under light protection. The percentage of apoptosis was confirmed in cytospin preparations, and >90% were apoptotic. All subjects gave their informed consent for inclusion before they participated in the study. The study was conducted in accordance with the Declaration of Helsinki, and the protocol was approved by the Ethics Committee of Institutional Review Board (Project number CAAE-12743219.6.0000.5149).

### Bone Marrow–Derived Macrophages (BMDMs) and Treatment

BMDMs were prepared, as previously described (Marim et al., 2010), with modifications. In brief, bone marrow was collected from tibias and femurs of BALB/c mice and washed with Dulbecco’s modified Eagle medium (DMEM) containing penicillin 100 units/ml and streptomycin 100 μg/ml, and the suspension obtained was then centrifuged for 5 min at 1,200 rpm. The pellet was resuspended with complete conditioned media for BMDM differentiation [DMEM with 20% heat-inactivated fetal bovine serum (FBS) and 30% L929 cell filtered supernatant media], seeded on tissue culture plates, and incubated at 37°C with 5% CO_2_. After 3 days, the medium was supplemented with additional complete conditioned media. On day 7, the supernatant was removed, and adherent macrophages were detached using a cell scraper and plated (2 × 10^5^ cells/well) in 96-well plates. BMDMs were treated with BCA (10 or 100 µM) for 24 h or pretreated with G15 (15 µM) and H89 (20 µM) for 1 h prior to treatment with BCA for further 24 h. Control cells were treated only with the vehicle (DMSO, 0.1%).

### Efferocytosis Assay *In Vitro*

*In vitro* efferocytosis was performed, as previously described (Budai et al., 2019; Vago et al., 2020). BMDMs were co-incubated with CFSE-labeled apoptotic thymocytes or neutrophils in a proportion of 3:1 (apoptotic cells/macrophages) for 1 h. After co-culture, apoptotic cells that had not been phagocytosed were removed by washing the wells with PBS 3 times. Efferocytosis by adherent macrophages was assessed by flow cytometric analyses using the frequency of F480^+^/CFSE^+^ cells, or as mean florescence intensity (MFI) of CFSE (flow cytometer laser set at 488). Doublets were determined and eliminated from data analysis.

### Histological Analysis

Tibiofemoral joint samples were collected and processed, as previously described (Queiroz-Junior et al., 2011). In brief, samples were fixed in 10% buffered formalin (pH 7.4), decalcified for 30 days in 14% EDTA, embedded in paraffin, sectioned (5 µm), and stained with hematoxylin and eosin (H&E). Two sections of knee joints were examined and scored by a single pathologist (CQ-J) in a blinded manner. The parameters evaluated were severity of synovial hyperplasia, intensity and extension of inflammatory infiltrates, presence of inflammatory cells in the synovial cavity, and vascular hyperemia. The grades were summed to obtain a histopathological score (ranging from 0 to 9).

### Statistical Analysis

The data and statistical analysis complied with the recommendations of the British Journal of Pharmacology on experimental design and analysis (Curtis et al., 2018). Studies were designed to generate groups of equal size, using randomization and blinded analysis. The group size selection for each protocol was also based on our previous studies. Data were tested for normality using the Shapiro–Wilk test, and statistical significance was determined using GraphPad Prism 8 software. All results are expressed as mean ± SEM. Data were analyzed by one-way ANOVA, followed by the Tukey posttest or Holm-Sidak’s when compared selected pairs of means based on experimental design. When only two groups were evaluated, Student’s t test was used. A value of *p* < 0.05 was considered significant.

## Results

### Biochanin A Reduces Neutrophil Accumulation, Mechanical Hypernociception, and Pro-Inflammatory Mediators of Arthritic Joints

**FIGURE 1:**
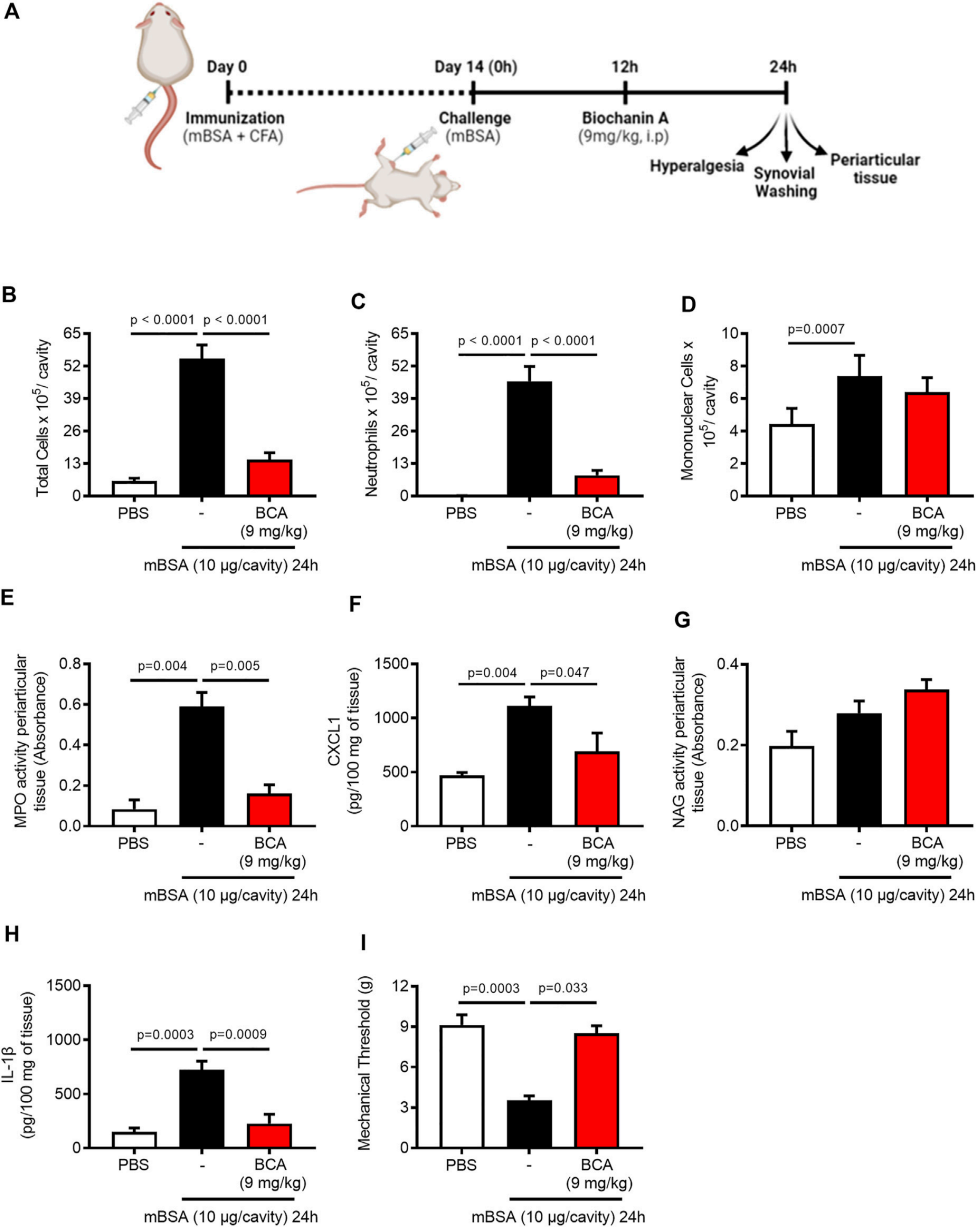
Biochanin A reduces neutrophil accumulation, mechanical hypernociception, and pro-inflammatory mediators of arthritic joints. Immunized mice received an injection of mBSA (10 μg/10 μL of PBS) into the tibiofemoral joint. Control mice were injected with PBS (10 μL). At the peak of inflammation (12 h post-mBSA challenge), mice were treated with an i.p. injection of BCA (9 mg/kg). Cells were harvested from the articular cavity at 24 h post-challenge (12 h after treatment) **(A)**. The number of total leukocytes **(B)**, neutrophils **(C)**, and mononuclear cells **(D)** were evaluated by counting cytospin. The activity of myeloperoxidase (MPO) and activity of N-acetylglucosaminidase (NAG) were evaluated in the periarticular tissue **(E**,**G**, respectively**)**. The levels of CXCL1 **(F)** and IL-1β **(H)** in the joint tissue homogenate were quantified by ELISA, and **(I)** mechanical hypernociception was recorded by the electronic von Frey algesimeter. The results are represented as mean ± SEM of five mice in each group. Significance was calculated using one-way ANOVA followed by Tukey’s test. The specified *p* = value is shown in the figure.

To study the effect of BCA (chemical structure is represented in Supplementary Figure S1A) on the inflammatory response and resolution, we used a well-established model of AIA characterized by an intense influx of leukocytes, predominantly neutrophils, which peaked from 12 to 24 h after the challenge with mBSA (Lopes et al., 2011). We have previously shown that the BCA pretreatment prevented the influx of neutrophils in a model of zymosan-induced arthritis (Felix et al., 2020). Here, the mice received an intra-articular injection of mBSA, and they were treated with BCA (0.36, 1.8, or 9 mg/kg) in the peak of neutrophil recruitment (12 h after challenge with antigens); the cells were recovered from the synovial cavity 24 h after the challenge to assess the accumulation of inflammatory cells (Figure 1A). BCA of a higher dose reduced the number of accumulated leukocytes (Supplementary Figure S1B), mostly neutrophils (Supplementary Figure S1C), with no change in the number of mononuclear cells (Supplementary Figure S1D). Of note, the higher dose promoted efficient reduction in the number of neutrophils in the cavity, and it was used for the next experimental procedures.

Consistent with the latter findings, BCA (9 mg/kg) efficiently caused a reduction in the number of total leukocytes (Figure 1B), and this reduction was due to the inhibition of neutrophil accumulation in the synovial cavity and periarticular tissue (Figures 1C,E, respectively) and reduced levels of CXCL1 (Figure 1F). However, BCA did not affect the accumulation of mononuclear cells in the synovial cavity (Figure 1D) and periarticular tissue (Figure 1G). In addition, BCA reduced the levels of IL-1β in the periarticular tissue (Figure 1H) and ameliorated mechanical hypernociception (Figure 1I), an index of pain and joint dysfunction, as measured by decrease in the paw withdrawal threshold.

### Biochanin A Resolves Neutrophilic Inflammation in the AIA Model

**FIGURE 2:**
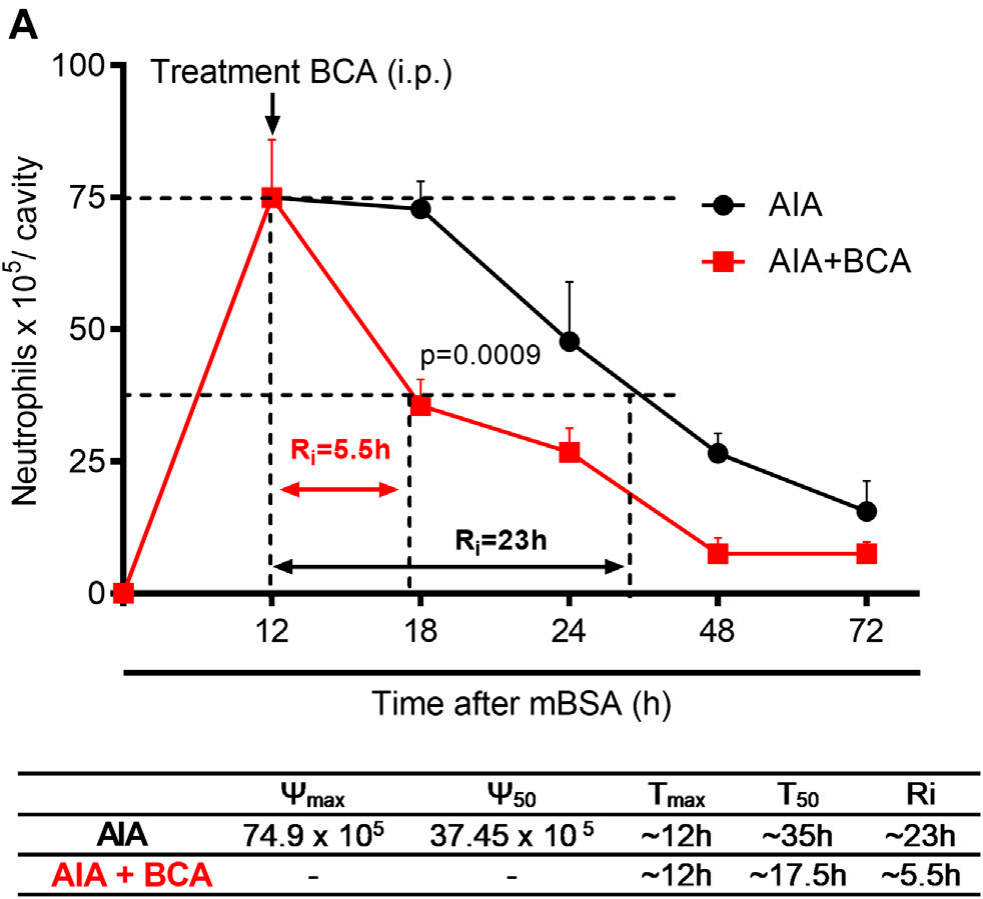
Treatment with biochanin A in antigen-induced arthritis induce timely resolution. Immunized mice received an injection of mBSA (10 μg/10 μL of PBS) into the tibiofemoral joint. Control mice were injected with PBS (10 μL). At the peak of inflammation (12 h post-mBSA challenge), mice were treated with an i.p. injection of BCA (9 mg/kg). Cells were harvested from the articular cavity at 12, 18, 24, 48, and 72 h after mBSA injection. Resolution indices were calculated by monitoring the neutrophil number in the synovial cavity **(A)**. Of note, T_max_ = 12 h, the time point when the neutrophil number reaches maximum; T_50_ BCA ∼17.5 h, the time point when the neutrophil number reduces to 50% of maximum; and resolution interval R_i_ BCA ∼5.5 h, the time period when 50% PMNs are lost from the articular cavity. The results are represented as mean ± SEM of five mice in each group. Significance was calculated using one-way ANOVA, followed by Tukey’s test. The specified *p* = value is shown in the figure.

Next, we investigated the effect of BCA on neutrophil infiltration kinetics to determine the time point that a 50% loss in neutrophil numbers occurred. To this end, resolution indices were calculated. These measurements are widely used to determine the pro-resolving abilities of several molecules and compounds (Navarro-Xavier et al., 2010). BCA was administered intraperitoneally at the peak of inflammation (12 h after antigen challenge), and cells were harvested from the synovial cavity 12, 18, 24, 48, and 72 h after the challenge. Treatment with BCA shortened the resolution interval by ∼ 23 h observed in vehicle-treated arthritic mice to ∼5.5 h, indicating that BCA accelerated the clearance of neutrophils from the synovial cavity (Figure 2A). Thus, 18 h was the checkpoint necessary for the resolution in AIA after treatment with BCA and was used for harvesting cells from the synovial cavity for subsequent analysis.

### Biochanin A Drives Resolution of Inflammation by Enhancing Neutrophil Apoptosis

**FIGURE 3:**
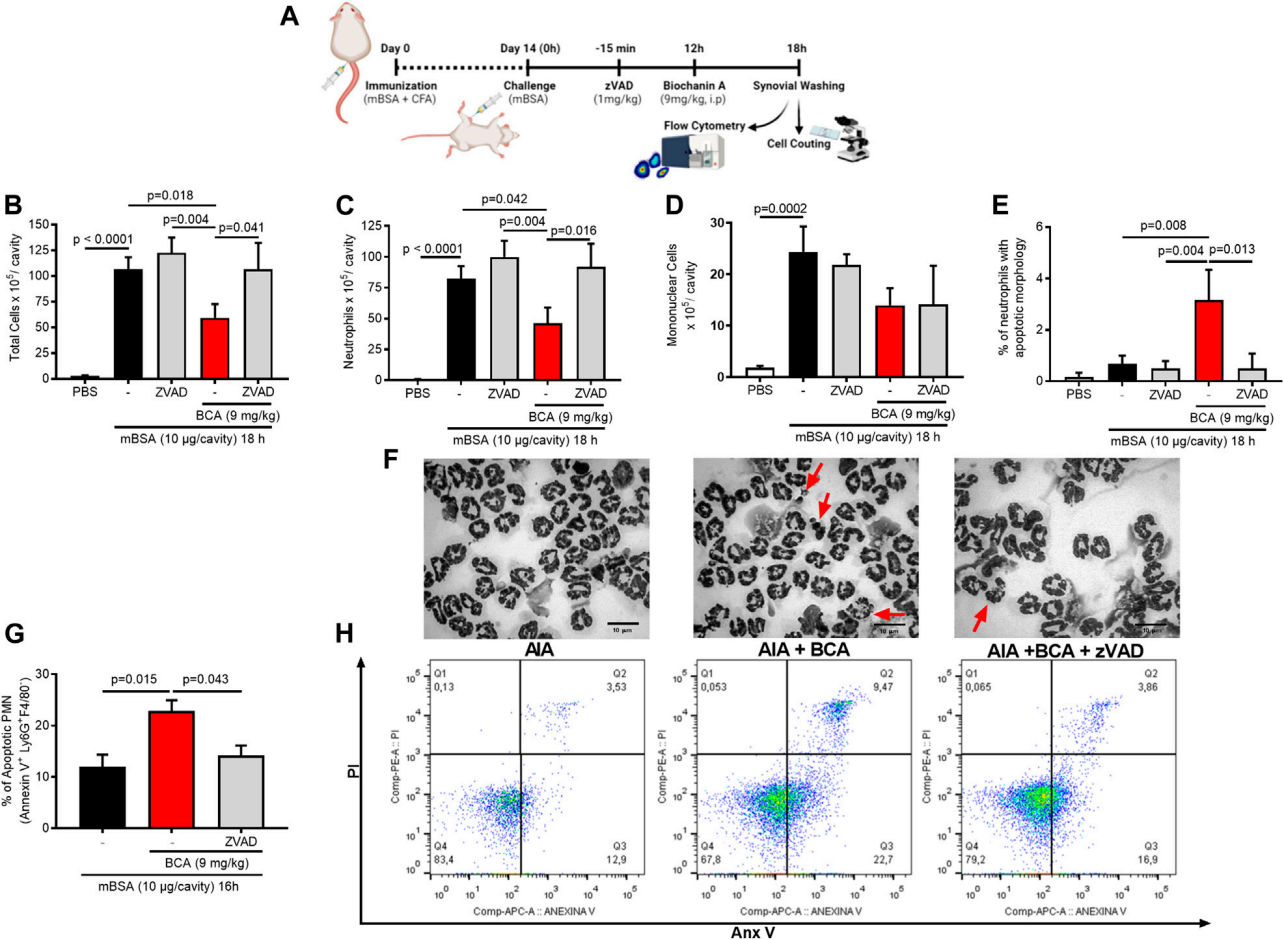
Biochanin A promotes resolution of the neutrophilic inflammatory response antigen-induced arthritis by inducing neutrophil apoptosis. Immunized mice received an injection of mBSA (10 μg/10 μL of PBS) into the tibiofemoral joint. Control mice were injected with PBS (10 μL). At the peak of inflammation (12 h post-mBSA challenge), mice were treated with an i.p. injection of BCA (9 mg/kg). The pan-caspase inhibitor zVAD-fmk (1 mg/kg, i.p.) was given 15 min before BCA. Cells were harvested from the articular cavity at 12 h post-challenge **(A)**. The number of total leukocytes **(B)**, neutrophils **(C)**, and mononuclear cells **(D)** was evaluated by counting cytospin. Cells with distinctive apoptotic morphology were evaluated 6 h after drug treatment and are expressed as percent of neutrophils with distinctive apoptotic morphology **(E)**. Representative figures of viable and apoptotic neutrophils (arrow) **(F)**. Magnification ×40. Apoptosis was biochemically evaluated 4 h after treatment BCA **(G)**, and the frequency of annexin V was determined by a flow cytometer **(H)**. The results are represented as mean ± SEM of five mice in each group. Significance was calculated using one-way ANOVA followed by Tukey’s test. The specified *p* = value is shown in the figure.

Next, we took an experimental approach to understand whether apoptosis induction was the mechanism by which BCA resolved neutrophilic inflammation in the AIA model. Mice were treated with BCA at the peak of inflammation (12 h), and 4 or 6 h later, the cells were harvested from the synovial cavity, and apoptosis was evaluated. The treatment with BCA decreased the number of total leukocytes (Figure 3B), and this reduction was due to the inhibition of neutrophils in the synovial cavity (Figure 3C), followed by an increase in the proportion of neutrophils with typical morphological apoptosis (Figures 3E,F). Then, to assess whether BCA-induced apoptosis was dependent on caspase activation, a broad-spectrum caspase inhibitor, zVAD-FMK, was used prior to treatment with BCA. As shown in Figures 3B–E, zVAD-FMK prevented the effects of BCA. There was no change in the number of mononuclear cells (Figure 3D). To confirm the increase in the proportion of apoptotic neutrophils, an apoptosis assay using labeled annexin V was performed by flow cytometry. BCA was able to increase the percentage of neutrophils positive for annexin V 4 h after treatment with BCA, and zVAD-FMK reduced this effect (Figures 3G,H). Importantly, zVAD-FMK alone does not affect leukocyte accumulation in the articular cavity after mBSA injection (Figures 3B–D). Taken together, these data showed that BCA promotes resolution of the neutrophilic inflammation by inducing caspase-dependent neutrophil apoptosis.

### Biochanin A Enhances the Efferocytic Ability of Murine Macrophages

**FIGURE 4:**
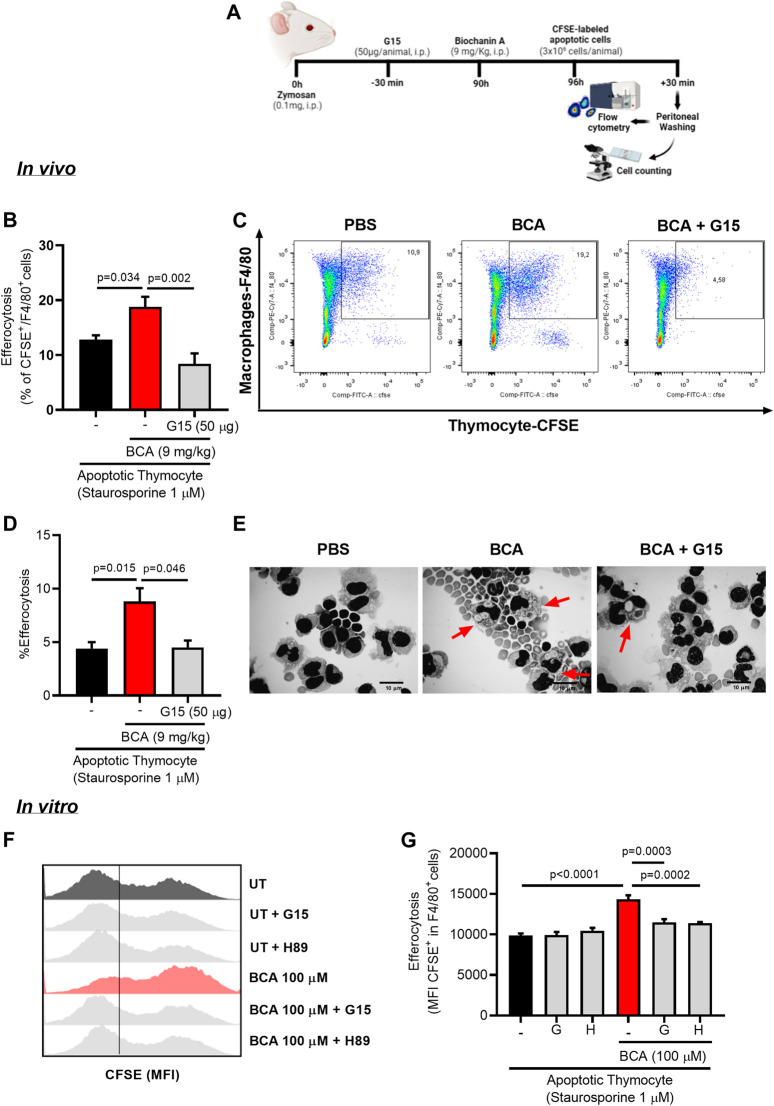
Biochanin A enhances efferocytic capacity of murine macrophage *in vivo* and *in vitro*. To determine efferocytosis *in vivo*, mice received an i.p. injection of 0.1 mg of zymosan and 90 h later were treated with an i.p. injection of BCA (9 mg/kg) or pretreated with G15 (50 µg/cavity, i.p.) for 30 min prior to treatment with BCA. Mice received an i.p. injection of 3 × 10^6^ apoptotic thymocyte labeled with fluorescent CFSE 6 h after treatment with BCA. The cells from the peritoneal cavity were collected 30 min later **(A)**. Efferocytosis was assessed by flow cytometry analyzing the frequency of double-positive cells for CFSE and F4/80 **(B)** and by counting cytospin slides **(D)**. Representative dot plots **(C)** and images of apoptotic thymocytes ingested by macrophages (arrows) are shown **(E)**. Magnification ×40. The *in vitro* efferocytosis assay was performed by co-culturing BMDMs with apoptotic thymocytes labeled with CFSE in a proportion of three thymocytes per macrophage. Macrophages were treated with BCA (100 µM) for 24 h or pretreated with G15 (G, 15 µM) or H89 (H, 20 µM) for 30 min prior to treatment with BCA for further 24 h. Efferocytosis was assessed by flow cytometry analyzing MFI (mean fluorescence intensity) of CFSE-labeled thymocyte in F4/80^+^ **(G)**. Representative histograms are shown in **(F)**. Flow cytometry data are expressed as MFI or frequency and are shown as the mean ± SEM of five mice in each group. Significance was calculated using one-way ANOVA followed by Tukey’s test. The specified *p* = value is shown in the figure.

One of the key determinants of inflammatory resolution is enhanced clearance of apoptotic neutrophils by efferocytosis. To understand whether BCA collaborating with inflammation resolution by affecting efferocytosis directly, we assessed efferocytosis using a protocol in which apoptotic cells are given as prey into the peritoneal cavity of the mice (Vago et al., 2019). For that, BALB/c mice received an intraperitoneal injection of zymosan (0.1 mg/cavity) to induce macrophage recruitment. After 90 h from zymosan injection, mice were treated intraperitoneally with BCA (9 mg/kg) and 6 h later received an intraperitoneal injection of CFSE-labeled apoptotic thymocytes. Cells were collected from the peritoneal cavity 30 min after the injection of apoptotic thymocytes and then labeled with F4/80 to analyze the presence of efferocytosis by flow cytometry and light microscopy (Figure 4A). The treatment with BCA improved the efferocytic capacity of peritoneal macrophages, as observed by the percentage of F4/80^+^/CFSE^+^ cells determined by flow cytometry (Figures 4B,C), or by counting the percentage of macrophages that had ingested apoptotic thymocytes, on cytospin slides (Figures 4D,E). This effect was entirely reversed by blockade of GPR30 with G15, a common target for isoflavones expressed in a wide variety of tissues, such as cartilage and bone (Ribeiro et al., 2020; Zhao et al., 2020; Figures 4B–E).

Our next step was to evaluate the impact of treatment with BCA on efferocytosis *in vitro*. Initially, BMDMs from BALB/c were treated with different concentrations of BCA (10 or 100 µM) for 24 h. BMDMs were co-cultured with CFSE-labeled apoptotic thymocytes (1:3, respectively) for 1 h. Then, the cells were marked with F4/80 and analyzed by flow cytometry. Treatment with BCA at a dose of 100 µM increased the engulfment of apoptotic thymocytes by BMDMs compared to untreated macrophages, as indicated by the higher MFI (mean fluorescence intensity of CFSE inside F4/80^+^ cells) (Supplementary Figures S2B,C). In another protocol, BMDMs were pretreated with G15 (15 µM) or H89 (20 µM) for 30 min prior to the treatment with BCA (100 µM). The engulfment of apoptotic thymocytes increased in BMDMs treated with BCA, as observed by MFI of CFSE in F4/80^+^ cells (Figures 4F,G), and this effect was prevented by GPR30 antagonism and PKA inhibition by G15 and H89, respectively (Figures 4F,G). Of note, we observed similar effects when using human neutrophils as prey (Supplementary Figures S2D,E and S3A,B). These data indicate that BCA improves macrophage efferocytosis, influencing directly on the clearance of apoptotic cells *in vivo* and *in vitro* in a GPR30/PKA-dependent mechanism.

### Biochanin A Resolves Neutrophilic Inflammation in the AIA Model in a GPR30/PKA-Dependent Manner

**FIGURE 5:**
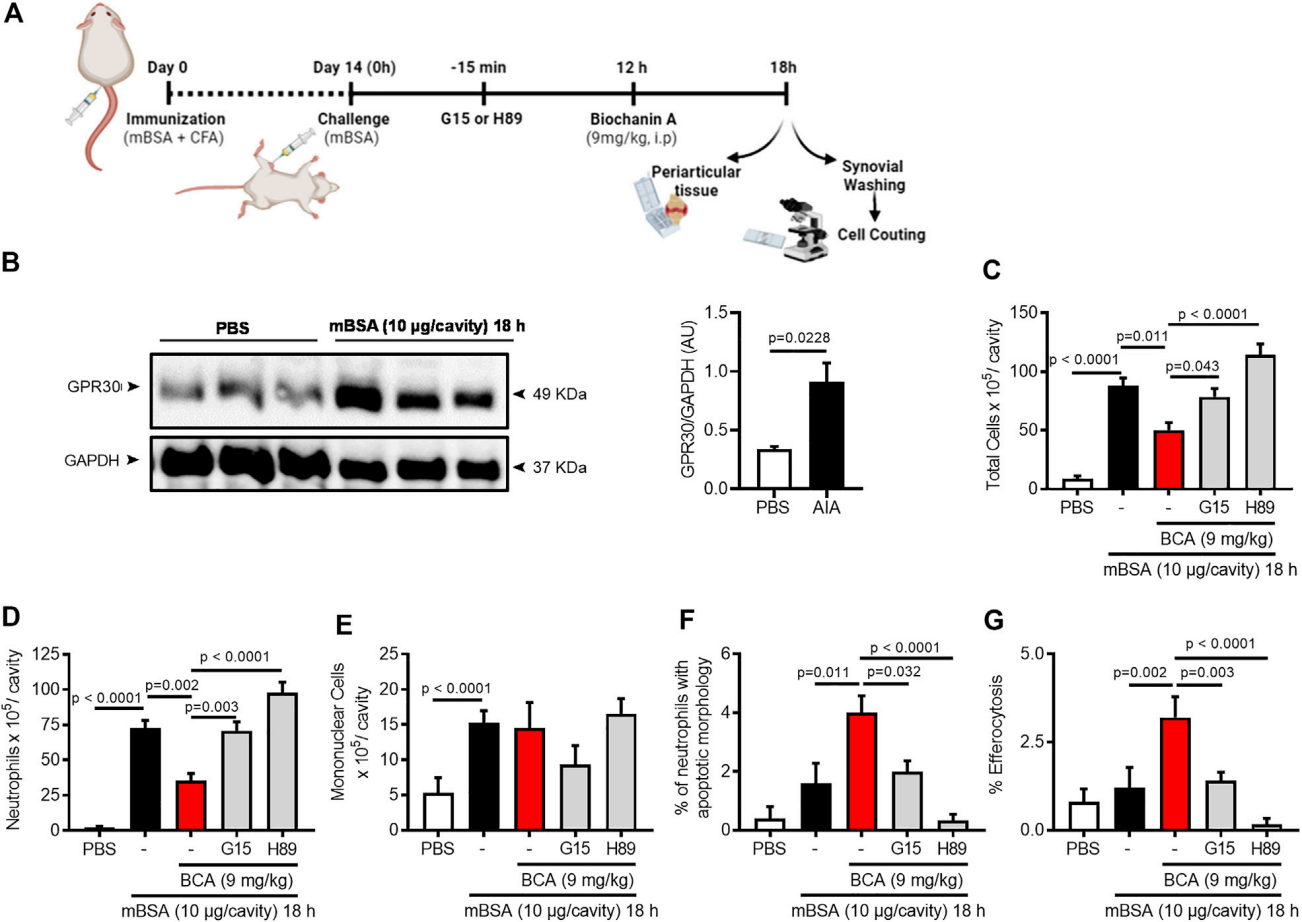
Biochanin A resolves neutrophilic inflammation in antigen-induced arthritis in a GPR30/PKA-dependent manner. Immunized mice received an injection of mBSA (10 μg/10 μL of PBS) into the tibiofemoral joint. Control mice were injected with PBS (10 μL). At the peak of inflammation (12 h post-mBSA challenge), mice were treated with an i.p. injection of BCA (9 mg/kg). G15 (50 µg), a GPR30-selective antagonist, and H89 (100 µg), a PKA inhibitor were given 15 min before BCA by intra-articular injection **(A)**. Periarticular tissue was collected 6 h after treatment for Western blot analysis for the detection of GPR30 **(B)**. For loading control, membranes were re-probed with anti-GAPDH. Cells were harvested from the articular cavity at 12 h post-challenge (6 h after treatment) for the count of the number of total leukocytes **(C)**, neutrophils **(D)** mononuclear cells **(E)**, cells with distinctive apoptotic morphology **(F),** and percentage of efferocytosis **(G)**. The results are represented as mean ± SEM of five mice in each group. Significance was calculated using one-way ANOVA followed by Tukey’s test or Holm-Sidak’s when comparing the AIA + BCA group with the AIA + BCA + G15 or AIA + BCA + H89 group. The specified *p* = value is shown in the figure.

Since BCA treatment improves macrophage efferocytosis in a GPR30/PKA-dependent mechanism, we intended to analyze the role of GPR30 and PKA in the AIA resolution induced by BCA treatment. First, we found that expression of GPR30 was higher in inflamed periarticular tissue (Figure 5B). Next, we investigated whether GPR30 was involved in the pro-resolving actions of the BCA in the AIA model. The treatment with BCA at the peak of inflammation (12 h after antigen challenge) decreased inflammation and promoted resolution of inflammation by decreasing the number of accumulated leukocytes (Figure 5C), mostly neutrophils in the synovial cavity when the cells were recovered 18 h after the challenge (Figure 5D), which was followed by an increased proportion of apoptotic neutrophils (Figure 5F) and enhanced efferocytosis (Figure 5G), two important steps for a proper resolution of inflammation.

Antagonism of GPR30 by treatment with a selective receptor antagonist, G15, 30 min before BCA prevented the pro-resolving actions of BCA (Figures 5C–G). In addition, we investigated whether PKA (a cAMP effector protein) was involved in pro-resolving actions of BCA *in vivo*. To this end, mice were pretreated with H89, a PKA inhibitor, 30 min before BCA. PKA inhibition reversed the effect of BCA on neutrophil accumulation, neutrophil apoptosis, and efferocytosis (Figures 5C–G), suggesting the participation of PKA in BCA-induced resolution. Noteworthy, there was no change in the number of mononuclear cells (Figure 5E). Furthermore, G15 or H89 alone did not modify the leukocyte recruitment into the articular cavity after mBSA injection (Supplementary Figures S4A–C).

### Biochanin A Reduces Joint Dysfunction and Mediators of Joint Inflammation in a GPR30/PKA-Dependent Mechanism

**FIGURE 6:**
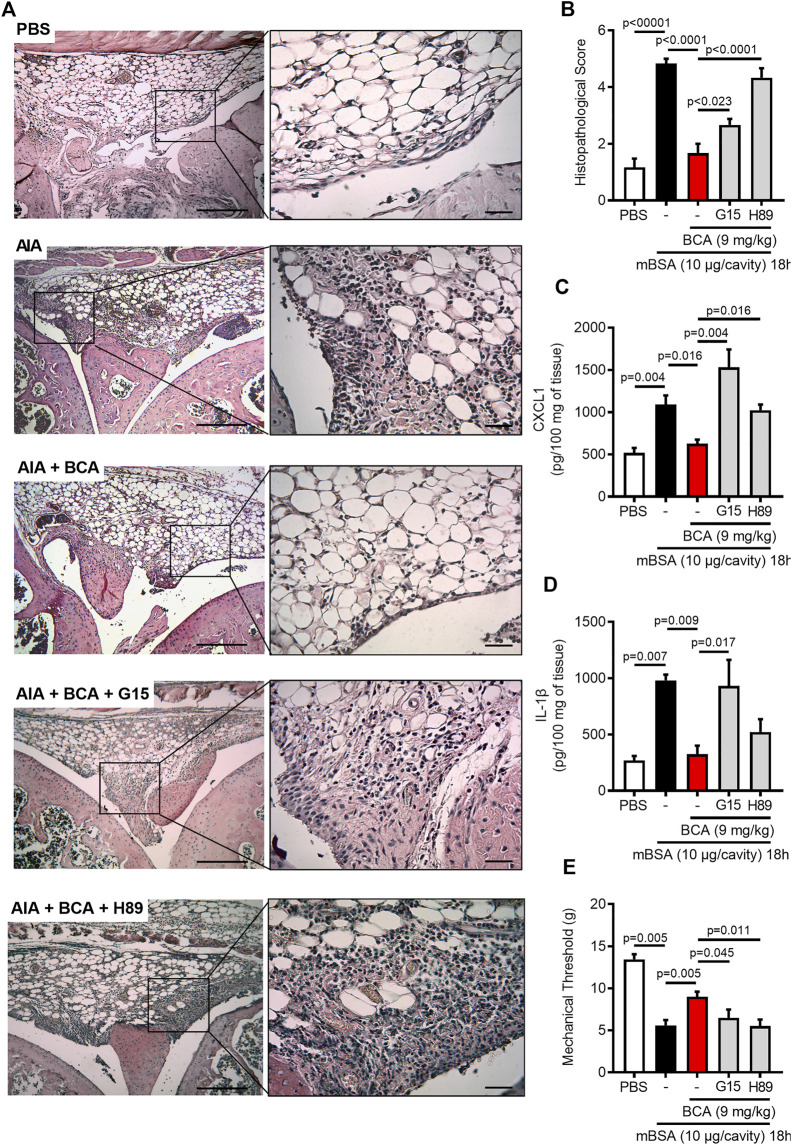
Biochanin A reduces damage tissue and hypernociceptive response in antigen-induced arthritis. Immunized mice received an injection of mBSA (10 μg/10 μL of PBS) into the tibiofemoral joint. Control mice were injected with PBS (10 μL). Representative histological slide to H&E of the knee from mice control (PBS), mBSA challenge (AIA), mice challenge with mBSA and post-treated with Biochanin A (AIA + BCA), mice challenge with mBSA posttreated with BCA or G15 or H89 at 15 min before BCA (AIA + BCA + G15 and AIA + BCA + H89, respectively) **(A)**. Histopathological score quantification of knee joint samples extracted 18 h after challenge **(B)**. Levels of CXCL1 **(C)** and IL-1β **(D)** in the joint tissue homogenate were evaluated by ELISA. The mechanical hypernociception **(E)** was recorded by an electronic von Frey algesimeter. The results are represented as mean ± SEM of five mice in each group. Significance was calculated using one-way ANOVA followed by Tukey’s test or Holm-Sidak’s when comparing the AIA + BCA group with the AIA + BCA + G15 or AIA + BCA + H89 group. The specified *p* = value is shown in the figure.

Having demonstrated the pro-resolving properties of BCA, we turned our attention to the effect of treatment with BCA on tissue damage of the joints and pain. The injection of mBSA induced synovial hyperplasia, vascular hyperemia, and infiltration of leukocytes in the synovium and articular cavity 18 h after antigen injection. The treatment with BCA ameliorated all those histological parameters (Figure 6A) and decreased the histopathological score (Figure 6B). Consistent with the pathology scoring and data of Figure 1, treatment of mice with BCA was also associated with decreased levels of CXCL1 and IL-1β in periarticular tissue (Figure 6C,D, respectively) and reduced hypernociception (Figure 6E) when inflammation was analyzed 18 h after antigen injection. Of note, the effects of BCA described above have been reversed by GPR30 antagonism and PKA inhibition (Figures 6A–E). Exceptionally, inhibition of PKA with H89 did not reverse completely the levels of IL-1β in periarticular tissue (Figure 6D). Overall, treatment with BCA promoted a reduction of mediators of joint inflammation, associated with ameliorated mechanical hypernociception and tissue architecture in a GPR30/PKA-dependent mechanism.

## Discussion

In this study, we investigated whether treatment with BCA at the peak of inflammation would be able to promote pro-resolving effects in a murine model of arthritis. Our major findings can be summarized as follows: treatment with BCA 1) led to a reduction in the accumulation of neutrophils in the synovial cavity, 2) anticipated the resolution index, 3) induced caspase-dependent neutrophil apoptosis and increased the efferocytosis, and 4) reduced the production of pro-inflammatory mediators, hypernociception, and joint pathology. Mechanistically, the pro-resolving effects of BCA were 5) dependent on the GPR30/PKA axis.

To date, BCA has been well-known for its anti-inflammatory effects. By reducing neutrophil recruitment and production of the inflammatory cytokines, BCA attenuated the development of zymosan-induced arthritis in mice (Felix et al., 2020), which correlates with the lower expression of adhesion molecules (VCAM-1, ICAM-1, and E-selectin) in neutrophils treated with BCA *in vitro* (Ming et al., 2015). However, effects of the BCA on the clearance of neutrophils have not been investigated yet. Of note, anti-inflammatory treatment controls inflammation by blocking pro-inflammatory mediators that are involved in the initial stage of inflammation, while pro-resolving therapies change the course of the established inflammatory response, shortening time and accelerating resolution (Perretti et al., 2015; Fullerton and Gilroy, 2016). Our data are the first to show that treatment with BCA when inflammation had already established resolves neutrophilic inflammation in AIA, mainly by increasing the apoptosis of neutrophils and subsequent efferocytosis, accelerating resolution (as demonstrated by reduction in Ri index).

Previous studies have established that BCA induces apoptosis in cancer cells via regulation of a variety of signaling molecules, such as activating the cleavage of caspase-3 and caspase-9, the release of cytochrome c (Hsu et al., 2018), and decreasing expression of anti-apoptotic proteins, B-cell lymphoma protein 2 (Bcl-2), and B-cell lymphoma extra-large (Bcl-xL) (Su et al., 2003; Mansoor et al., 2011; Puthli et al., 2013). Recently, we showed that BCA induces neutrophil apoptosis *in vitro* (Felix et al., 2020). In the current study, we demonstrated that the reduction in the accumulation of neutrophils in the arthritic synovial cavity was associated with the apoptosis of these cells and that this mechanism was caspase-dependent, as confirmed by the reduction of apoptosis by treatment with a pan-caspase inhibitor, zVAD-fmk. The caspase inhibitor reduced apoptosis and consequently prevented the pro-resolving effects of BCA in the AIA model. It is well-described that apoptosis of effector leukocytes (mainly neutrophils) represents an important step toward a successful inflammation resolution of acute inflammation (Hallett et al., 2008; Sousa et al., 2009; Vago et al., 2012). Importantly, the induction of apoptosis by BCA appears to be specific for neutrophils in the AIA model, since no change in the number of mononuclear cells in treated arthritic animals was observed. *In vitro* studies have shown that BCA has no effect on the viability of RAW 264.7 murine cell line (Lam et al., 2004; Kole et al., 2011). Monocytes differentiate into macrophages in the inflamed site and represent an important actor during the resolution of inflammation, by promoting the clearance of apoptotic neutrophils and the secretion of pro-resolving mediators (Greenlee-Wacker, 2016). To the best of our knowledge, a characterization of macrophage repertoire after treatment with BCA has not been performed and still requires full characterization to better understand its role in the resolution process induced by BCA.

Apoptosis is a process synchronized by interconnection and the removal of apoptotic bodies by macrophages (efferocytosis) (Boada-Romero et al., 2020), both events necessary to lead a complete and effective resolution. Efferocytosis involves the recruitment of macrophages, by apoptotic particles issued as a find-me signal and recognition of phosphatidylserine exposed on the apoptotic cell surface as an eat-me signal for cell engulfment by macrophages in a plasma membrane–derived vacuole called the “efferosome,” and these efferosomes gradually mature to provide the degradative enzymes required to digest apoptotic bodies, triggering an anti-inflammatory and nonimmunogenic post-engulfment signaling (Elliott et al., 2017; Lin et al., 2020). Thus, defective removal of apoptotic cells resulting from impaired efferocytosis can lead to tissue dysfunction and inflammatory and infectious diseases, including lung disease, arthritis, and atherosclerosis (Doran et al., 2020; Zheng et al., 2021). A recent study has shown that treatment with 2-hydroxybenzylamine (2-HOBA), a compound found naturally in buckwheat and in nutritional supplement, promotes efferocytosis of apoptotic cells in atherosclerotic plaques and contributes to reducing the inflammation and the development of atherosclerosis in mice (Tao et al., 2020). Previous studies have also shown that flavonoids can contribute to an efficient efferocytosis *in vitro* (Yen et al., 2014; Lai et al., 2018). Consistent with these reports, our data clearly show that treatment with BCA enhances macrophage efferocytic capacity both *in vitro* and in the joint cavity of arthritic mice, leading to the resolution of inflammation. Overall, the findings of this study revealed that treatment with BCA during an established inflammatory response can control inflammation and more than that activates key steps that drive to the resolution of neutrophilic inflammation. These data improve our understanding of the effects of BCA on the inflammatory response and highlight promising actions by which isoflavones can activate or accelerate pro-resolving programs. However, further studies are necessary to clarify whether the effects of BCA would contribute to additional pro-resolving mechanisms, such as the production of pro-resolving mediators.

GPR30 is a Gs-coupled seven-domain transmembrane protein reported to be activated with high affinity for estrogen and G1, a specific agonist for GPR30 (Filardo and Thomas, 2005; Revankar et al., 2005). Apart from these ligands, the flavonoids or phenolic phytochemicals, such as genistein, resveratrol, prunetin, quercetin, daidzein, and apigenin have been reported to exert actions via GPR30 (Khan et al., 2015; Prossnitz and Arterburn, 2015; Molina et al., 2018; Zhao et al., 2020). GPR30 activation has been shown to mediate anti-inflammatory protective effects in neuroinflammation (Guan et al., 2017), vascular inflammation (Chakrabarti and Davidge, 2012), and asthma (Itoga et al., 2015) and decreased the expression of TLR4 in murine macrophages induced by LPS (Rettew et al., 2010). Although GPR30 has been documented to be present constitutively in human neutrophils (Rodenas et al., 2017), its regulatory role in the neutrophilic inflammatory response and resolution has never been reported before. In the current study, we clearly show that GPR30 is upregulated in the periarticular tissue during synovial inflammation. More important, the pro-resolving effects of BCA mediated by GPR30 was totally reversed by the treatment with G15, a selective GPR30 antagonist (Dennis et al., 2009), suggesting a direct involvement of a specific GPR30-dependent mechanism in the resolution of inflammation induced by BCA in AIA.

Several lines of evidence demonstrated that GPR30 activation by agonists induces stimulation of adenylyl cyclase activity and cAMP elevation, and its signaling triggered the cAMP-dependent PKA pathway (Khan et al., 2015; Liu et al., 2018). The cAMP is a second intracellular messenger produced by the activity of adenylate cyclase that converts ATP to cAMP (Kamenetsky et al., 2006; Raker et al., 2016). It is known that levels of cAMP are regulated by phosphodiesterases (PDEs) (Conti and Beavo, 2007), enzymes that promote the hydrolysis of cAMP to AMP. PKA is well-known as the most important effector molecule of cAMP and interaction between these molecules promotes functional rearrangement with enzymatic activity (Cheng et al., 2008). Several studies described that cAMP-PKA signaling is involved in key steps to drive effective inflammation resolution (Sousa et al., 2009, 2010; Tavares et al., 2020), and the inhibition of PKA with H89 impairs cAMP-mediated resolution in a model of LPS-induced inflammation (Lima et al., 2017; Negreiros-Lima et al., 2020). Our findings reinforce those of the literature since H89 reverted resolution induced by BCA in the AIA model. In agreement, some studies have been suggested an interplay between BCA and PKA/cAMP signaling pathway activation. A recent study demonstrated the inhibition of PKA with H89 reduced relaxation in the coronary artery induced by BCA (Kumar et al., 2020). Furthermore, another study demonstrated that BCA promotes increased levels of cAMP by selective inhibition of PDE4 and suppresses ovalbumin-induced airway hyperresponsiveness (Ko et al., 2011). Altogether, these data led us to conclude that the GPR30/PKA/cAMP axis is involved in pro-resolving abilities induced by BCA observed in our model.

Joint pain is a marked symptom in arthritic patients associated with loss of function (Walsh and McWilliams, 2014). The induction and maintenance of joint pain are associated with intense CXCL1-mediated neutrophil influx and production of hyperalgesic mediators, such as IL-1β (Cunha et al., 2010; Sachs et al., 2011; Amaral et al., 2012), and blockade these mediators have been described to suppressed joint pain and damage in murine model of arthritis (Williams et al., 2000). In addition to joint pain, neutrophils are the main responsible for tissue damage (Fattori et al., 2016). Previous studies have shown that BCA potentially inhibit pro-inflammatory cytokines production including TNF-α and IFN-γ in neutrophils (Felix et al., 2020) and IL-6 and IL-1β in macrophages (Kole et al., 2011; Qiu et al., 2012). Furthermore, BCA has been described to inhibit neuropathic pain in diabetic rats (Chundi et al., 2016). Importantly, BCA ameliorated cartilage degradation during the progression of osteoarthritis in rabbit (Wu et al., 2014). In agreement with these previous studies, we demonstrated that treatment of arthritic mice with BCA reduced the secretion of CXCL1 and IL-1β in the periarticular tissue, and this effect was correlated with reduction in the number of neutrophils in synovial cavity, reduction of tissue damage and pain. More importantly, GPR30 blockage and PKA inhibition prevented the effects of BCA, reinforcing the direct involvement of the role of GPR30/PKA to inflammation resolution induced by BCA in AIA model.

**FIGURE 7:**
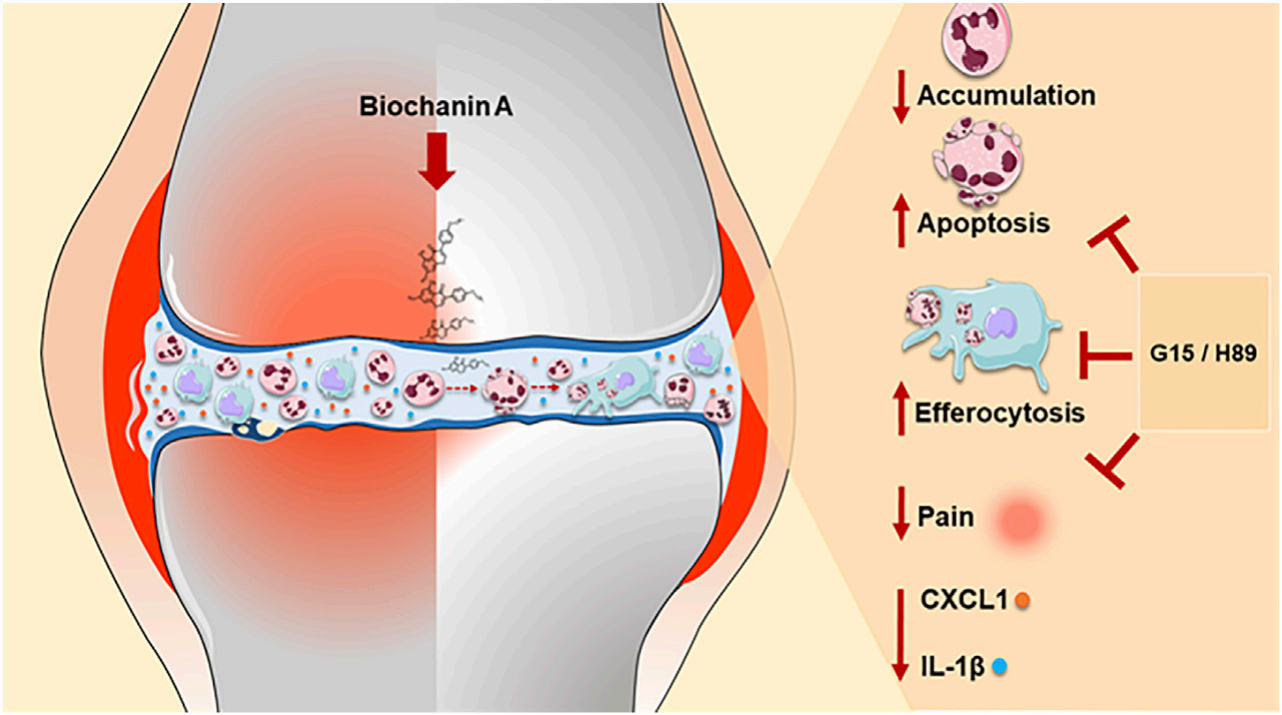
Schematic representation of the effects of BCA on the resolution of neutrophilic resolution in antigen-induced arthritis. Our results indicate that treatment with BCA led to a reduction in the accumulation of neutrophils in the synovial cavity in response to mBSA injection, induced caspase-dependent neutrophil apoptosis, increased the efferocytosis and anticipated the resolution index. In addition, reduction of pro-inflammatory mediators (CXCL1 and IL-1β levels), decreased hypernociception and consequently restoration of tissue damage induced by mBSA. Mechanistically, the pro-resolving effects of BCA were dependent on the GPR30/PKA signaling pathway, as demonstrated by blocking of GPR30 with G15 and inhibition of PKA with H89, which affects actions of BCA. The pro-resolving effects of BCA summarized here provide evidence for the first time that treatment with BCA notably regulates key steps of resolution of inflammation.

Collectively, we provided evidence for the first time that treatment with BCA notably regulates key steps of resolution of inflammation by regulating neutrophil accumulation, inducing neutrophil apoptosis, decreasing cytokine release, and promoting efferocytosis in a GPR30/PKA-dependent manner (summarized in Figure 7). Thus, BCA may represent a potential therapeutic strategy in a neutrophilic inflammatory response.

## Data Availability

The original contributions presented in the study are included in the article/Supplementary Material, further inquiries can be directed to the corresponding author.

## Supplementary Material

SUPPLEMENTARY FIGURE 1 | Dose-response of treatment with Biochanin A in antigen-induced arthritis. Immunized mice received an injection of mBSA (10 μg/10 μl of PBS) into the tibiofemoral joint. Control mice were injected with PBS (10 μl). At the peak of inflammation (12 h post-mBSA challenge), mice were treated with an i.p. injection of BCA, at doses of 0.3, 1.8 or 9 mg/kg. Chemical structure of BCA is represented **(A)**. Cells were harvested from the articular cavity at 24 h post-challenge (12 h after treatment). The number of total leukocytes **(B)**, neutrophils **(C)** and mononuclear cells **(D)** were evaluated by counting cytospin. The results are represented as mean ± SEM of five mice in each group. Significance was calculated using one-way ANOVA followed by Tukey's test. The specified p=value is shown in the figure.

SUPPLEMENTARY FIGURE 2 | Effect of treatment with on efferocytosis *in vitro*. The in vitro efferocytosis assay was performed by co-culturing BMDMs with thymocyte or human neutrophil apoptotic labeled with CFSE in a proportion of 3 cells apoptotic per macrophage. Macrophages were treated with BCA (10 or 100 μM) for 24 h **(A)**. Efferocytosis was assessed by flow cytometry analyzing MFI (mean fluorescence intensity) of CFSE-labeled cells apoptotic in F4/80^+^ **(B,D)**. Representative histograms are shown in **(C,E)**. Flow cytometry data are expressed as MFI and are shown as the mean ± SEM. Significance was calculated using one-way ANOVA followed by Tukey's test. The specified p=value is shown in the figure.

SUPPLEMENTARY FIGURE 3 | Effect of treatment with BCA on macrophages efferocytosis *in vitro*. The in vitro efferocytosis assay was performed by co-culturing BMDMs with human neutrophil apoptotic labeled with CFSE in a proportion of 3 cells apoptotic per macrophage. Macrophages were treated with BCA (100 μM) for 24 h or pre-treated with G15 (G, 15μM) or H89 (H, 20 μM) for 30 min prior to treatment with BCA for further 24 h **(A)**. Efferocytosis was assessed by flow cytometry analyzing the frequency of double-positive cells for CFSE and F4/80 and representative dot plots are shown **(B,C)**. Flow cytometry data are expressed as frequency and are shown as the mean ± SEM. Significance was calculated using one-way ANOVA followed by Tukey's test. The specified p=value is shown in the figure.

SUPPLEMENTARY FIGURE 4 | Effects of treatment with 15 and H89 in antigen-induced arthritis. Immunized mice received an injection of mBSA (10 μg/10 μl of PBS) into the tibiofemoral joint. Control mice were injected with PBS (10 μl). At the peak of inflammation (12 h post-mBSA challenge), mice were treated with G15 (50 μg), a GPR30 selective antagonist, and H89 (100 μg), a PKA inhibitor BCA by intra-articular injection. Cells were harvested from the articular cavity at 18 h post-challenge. The number of total leukocytes **(A)**, neutrophils **(B)** and mononuclear cells **(C)** were evaluated by counting cytospin. The results are represented as mean ± SEM of five mice in each group. Significance was calculated using one-way ANOVA followed Tukey's. The specified p=value is shown in the figure.
