# Supplementary figures and images for: Biochanin A Regulates Key Steps of Inflammation Resolution in a Model of Antigen-Induced Arthritis via GPR30/PKA-Dependent Mechanism
Source: Front Pharmacol. 2021 Apr 26;12:662308. doi: 10.3389/fphar.2021.662308 (PMC8114065; doi:10.3389/fphar.2021.662308)

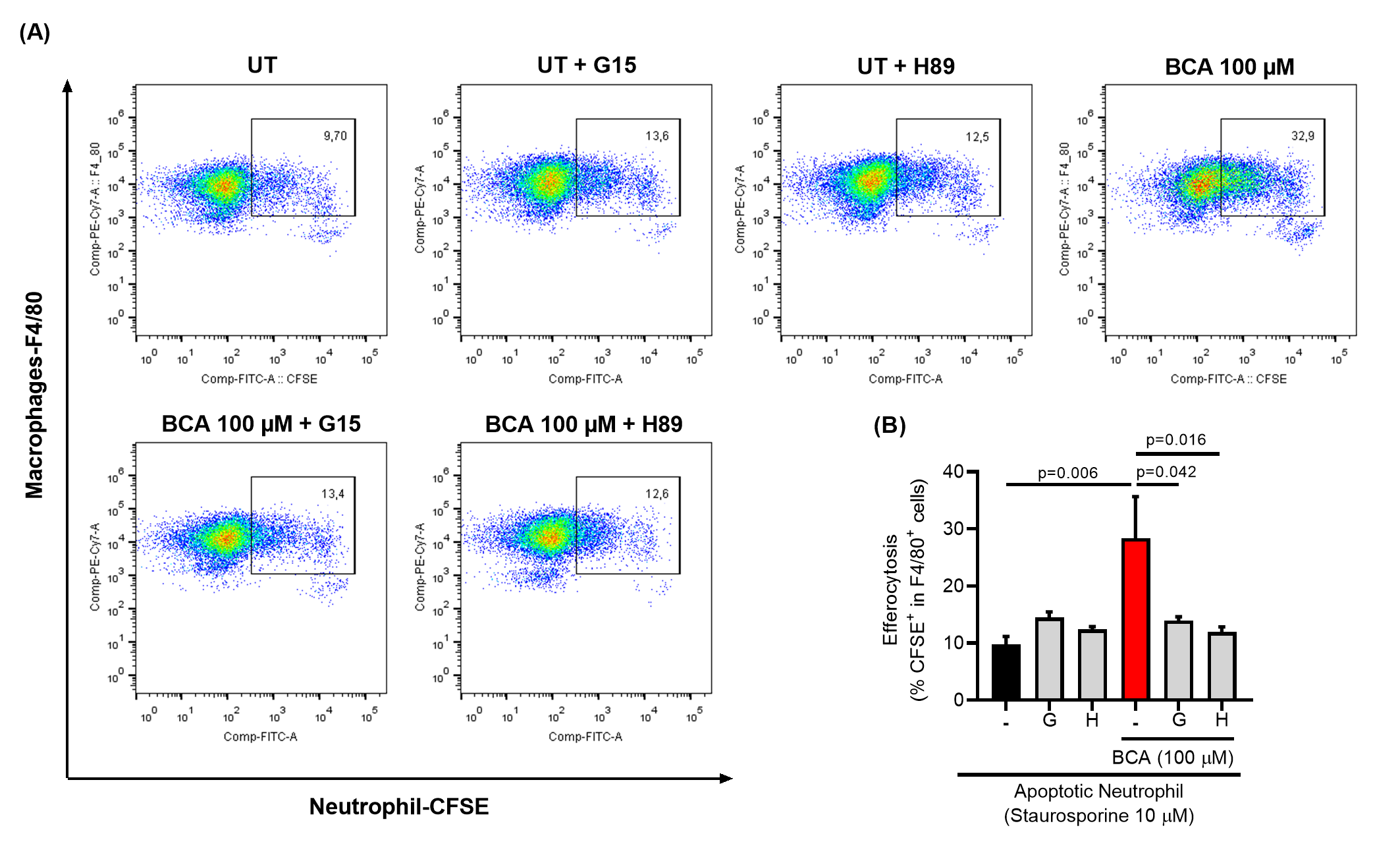

Supplement: Supplementary file 1 [file Image3.TIF]

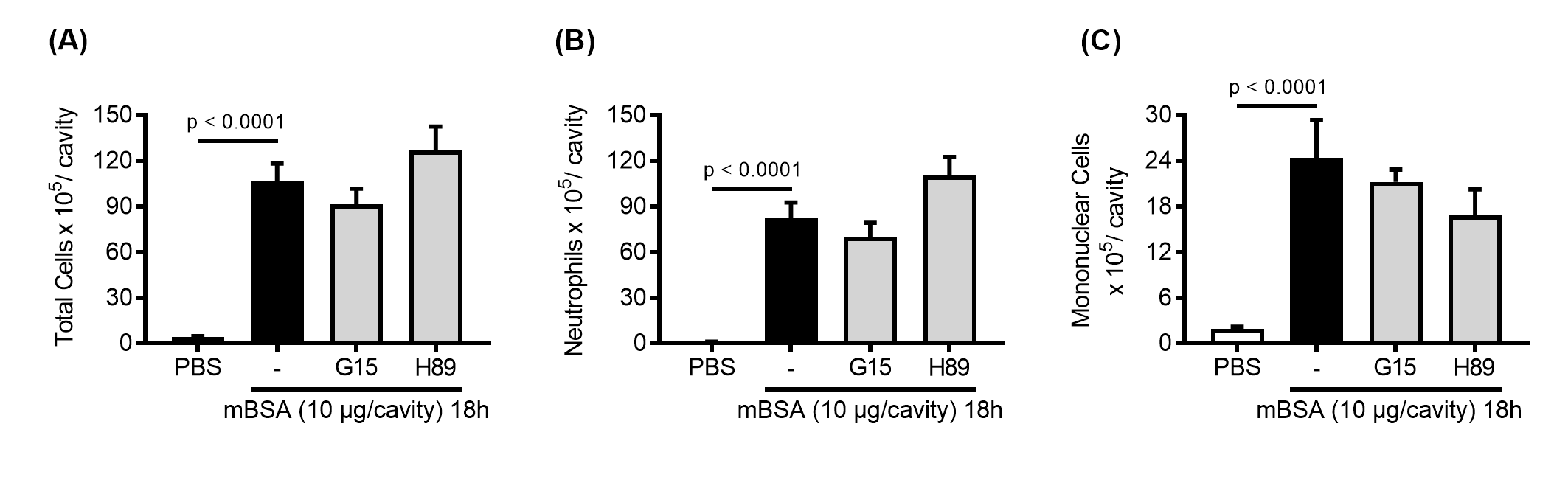

Supplement: Supplementary file 2 [file Image4.TIF]

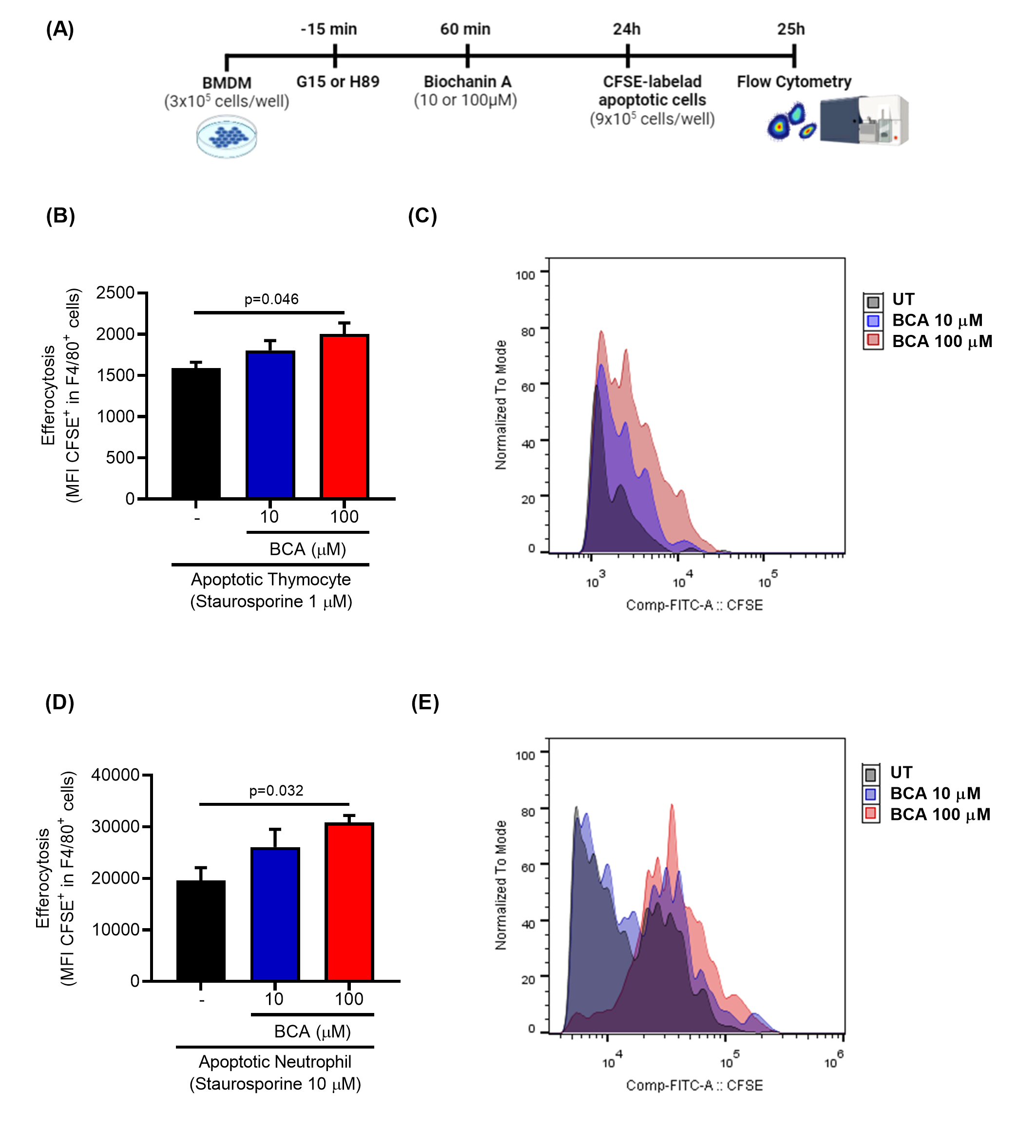

Supplement: Supplementary file 3 [file Image2.TIF]

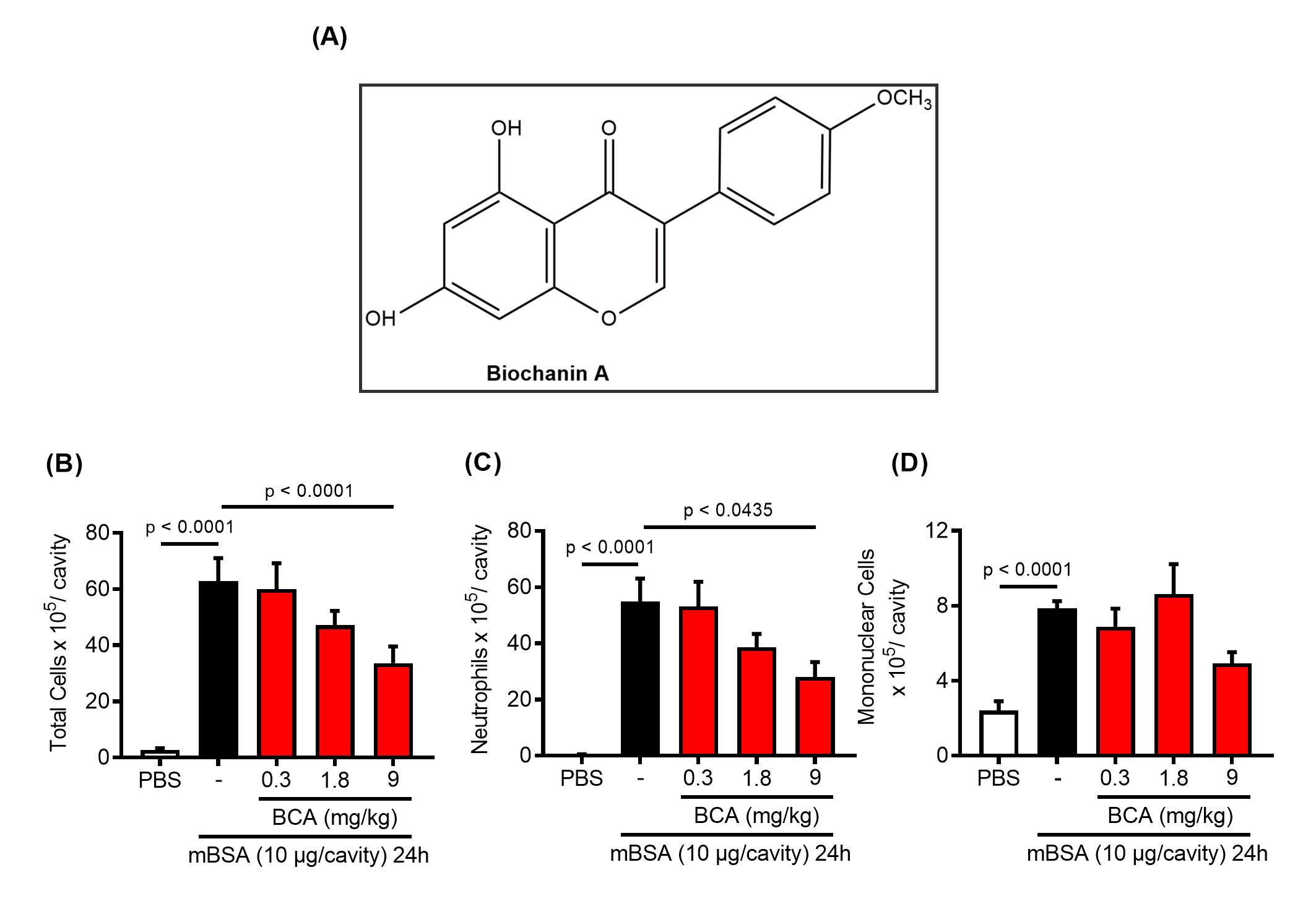

Supplement: Supplementary file 4 [file Image1.TIF]
